# Demography and rapid local adaptation shape Creole cattle genome diversity in the tropics

**DOI:** 10.1111/eva.12641

**Published:** 2018-05-18

**Authors:** Daniel Pitt, Michael W. Bruford, Mario Barbato, Pablo Orozco‐terWengel, Rodrigo Martínez, Natalia Sevane

**Affiliations:** ^1^ School of Biosciences Cardiff University Cardiff UK; ^2^ Sustainable Places Research Institute Cardiff University Cardiff UK; ^3^ Institute of Zootechnics Università Cattolica del Sacro Cuore Piacenza Italy; ^4^ Centro de investigaciones Tibaitatá Corporación Colombiana De Investigación Agropecuaria (Corpoica) Bogotá Colombia

**Keywords:** *Bos primigenius taurus*, Criollo, demographic history, *GDNF*, selection signatures, slick hair coat

## Abstract

The introduction of Iberian cattle in the Americas after Columbus’ arrival imposed high selection pressures on a limited number of animals over a brief period of time. Knowledge of the genomic regions selected during this process may help in enhancing climatic resilience and sustainable animal production. We first determined taurine and indicine contributions to the genomic structure of modern Creole cattle. Second, we inferred their demographic history using approximate Bayesian computation (ABC), linkage disequilibrium (LD) and *N*_e_ Slope (NeS) analysis. Third, we performed whole genome scans for selection signatures based on cross‐population extended haplotype homozygosity (XP‐EHH) and population differentiation (*F*_ST_) to disentangle the genetic mechanisms involved in adaptation and phenotypic change by a rapid and major environmental transition. To tackle these questions, we combined SNP array data (~54,000 SNPs) in Creole breeds with their modern putative Iberian ancestors. Reconstruction of the population history of Creoles from the end of the 15th century indicated a major demographic expansion until the introduction of zebu and commercial breeds into the Americas ~180 years ago, coinciding with a drastic *N*_e_ contraction. NeS analysis provided insights into short‐term complexity in population change and depicted a decrease/expansion episode at the end of the ABC‐inferred expansion, as well as several additional fluctuations in *N*_e_ with the attainment of the current small *N*_e_ only towards the end of the 20th century. Selection signatures for tropical adaptation pinpointed the thermoregulatory slick hair coat region, identifying a new candidate gene (*GDNF*), as well as novel candidate regions involved in immune function, behavioural processes, iron metabolism and adaptation to new feeding conditions. The outcomes from this study will help in future‐proofing farm animal genetic resources (FAnGR) by providing molecular tools that allow selection for improved cattle performance, resilience and welfare under climate change.

## 1 INTRODUCTION

Until recently, selection has occurred at a relatively slow rate in cattle and has been largely passive, driven by adaptations to diseases, dietary variation and local climatic patterns (Russell, 2007). After the domestication of cattle ~7,000–10,000 years ago (YA; Bruford, Bradley, & Luikart, 2003), farmers started to artificially breed animals with preferred phenotypes, although it was not until ~200 YA that European farmers began the formation of closed herds which developed into modern breeds (Taberlet, Coissac, Pansu, & Pompanon, 2011). However, another type of human endeavour has forced even higher selective pressures on a limited number of domestic animals concentrated in a brief period of time: long distance transportation, one example of which is the introduction of Iberian livestock species in the Americas. After the first arrival of cattle on the tropical Caribbean island Hispaniola in 1493, Creole livestock started to evolve into distinct ecotypes specifically adapted to a variety of environments and production systems. From this location, as well as reinforcements from Iberia and the Atlantic archipelagos during the 16th century, cattle populations expanded and spread throughout the Americas (Villalobos Cortés, Martinez, Vega‐Pla, & Delgado, 2009), starting from an estimated founding stock below 1,000 individuals (Rodero, Rodero, & Delgado, 1992). Introductions of northern European cattle into North America were also reported between 1608 and 1640 (Felius et al., 2014). After three centuries featuring the predominance of Creole cattle, population declines started with the introduction of other cattle around the middle of the 19th century, better suited to more intensive production and breeding systems (Willham, 1982). The introduction of European breeds (poorly adapted to the tropics but normally highly productive) and zebus (highly adapted to the tropics, but normally not as productive) resulted in the substitution of Creoles by a series of less adapted, admixed or commercial populations, displacing them into marginal areas.

Reconstructing the demographic history of Creole populations is therefore key to disentangling American livestock colonization dynamics and can contribute to a better understanding of the genomic signatures of breed evolution. Additionally, ongoing climate change is likely to lead to reductions in animal production and welfare in the future, which makes an understanding of the genomic regions selected under the major and rapid environmental changes imposed on Creole cattle, a useful tool for enhancing resilience and sustainable production in the short term. Therefore, the aims of this study were first to determine the contributions of different taurine and indicine ancestors on the genomic make‐up of Creole cattle. Our second aim was to infer the demographic history of Creole cattle populations by combining different approaches to investigate trends in effective populations size (*N*_e_): approximate Bayesian computation (ABC; Wegmann, Leuenberger, Neuenschwander, & Excoffier, 2010); linkage disequilibrium (LD) structure (SNeP; Barbato, Orozco‐terWengel, Tapio, & Bruford, 2015); and *N*_e_ Slope analysis (NeS). Finally, our third aim was to perform a whole genome scan for the signatures of selection based on cross‐population extended haplotype homozygosity tests (XP‐EHH; Sabeti et al., 2007) and population differentiation (*F*_ST_; Wright, 1949). To tackle these questions, we combined SNP array data in modern Creole cattle with modern day samples from breeds comprising their putative Iberian ancestors. By identifying genomic regions responding to these selection pressures, we aimed to provide valuable tools for improving cattle resilience, performance and welfare under climate change.

## 2 MATERIALS AND METHODS

### 2.1 Cattle populations and SNP array data

The data set comprised SNP array data from 412 individuals genotyped using the Illumina BovineSNP50 array versions 1 and 2, and the Bovine High Density BeadChip (Bovine Hapmap et al., 2009; Decker et al., 2009, 2014; Gautier, Laloë, & Moazami‐Goudarzi, 2010; Upadhyay et al., 2017; Supporting Information Table S1). Twenty‐nine animals were newly genotyped using the Illumina BovineSNP50 version 2 and Geneseek Genomic Profiler Bovine 150k (Supporting Information Table S1). We included six Creole populations adapted either to tropical humid (three Colombian breeds: Costeño con Cuernos, Romosinuano, San Martinero; a North American breed: Florida Cracker; and a Caribbean breed sampled in Brazil: Senepol) or dry conditions (Texas Longhorn). We also analysed the main breeds comprising their putative Iberian ancestors: (i) six different Lidia lineages, a breed that has not been selected for productivity traits and may be the most representative modern descendent of Iberian cattle herds back in the 15th century, retaining high genetic variability among lineages; (ii) Mostrenca, Retinta, Berrenda en Colorado, Cárdena Andaluza and Pajuna breeds, distributed throughout central and southern Iberia; and (iii) Asturiana de los Valles and Cachena, reflecting the northern Iberian genomic pool. The remaining breeds represent a hypothesized African taurine influence on Creole cattle (Baoule, Lagune, N’Dama, Somba; Miretti, Dunner, Naves, Contel, & Ferro, 2004), representatives of commercial European stock introduced to the Americas around the middle of the 19th century (Angus, Red Poll, Holstein, Jersey, Shorthorn) and potential indicine introgression into Creole cattle from tropical areas (Brahman, Nelore, Gir). SNP array data were merged, and those SNPs detected as triallelic were flipped using PLINK 1.90 (Chang et al., 2015; Purcell et al., 2007). The data set was then phased with Beagle 3.3.2 (Browning & Browning, 2007) and the genomic positions for each SNP mapped to the UMD3.1 bovine assembly (RefSeq:GCF_000003055.5). Only autosomal SNPs with a minor allele frequency (MAF) above 1% and a call rate of at least 90% across all breeds were retained for downstream analyses, leaving 33,342 SNPs.

### 2.2 Estimation of autosomal ancestry proportions and population divergence in Creole cattle

To determine the relative contribution of different potential taurine and indicine ancestors on the genomic structure of Creole cattle, population admixture analysis was carried out using the software Admixture v1.3 (Alexander, Novembre, & Lange, 2009) with 2,000 bootstraps for eight population clusters (K), corresponding to the African (two clusters), Iberian, Angus, Shorthorn, Holstein and Jersey taurine ancestries, as well as the Asian zebu ancestry. For this analysis, autosomal SNP array data were further pruned for LD higher than 0.1 using a sliding window approach of 50 SNPs and a step size of 10 SNPs. The results were graphically displayed using the POPHELPER R package (Francis, 2017).

Multidimensional scaling (MDS) was implemented using Hamming distances across 20 dimensions using PLINK. The first two (major) axes were visualized using R (R Core Team 2014). A Reynolds’ distance matrix was estimated between population pairs using Arlequin v3.5 (Excoffier & Lischer, 2010), and a neighbour‐net tree was constructed in SplitsTree v.4.14.4 (Huson & Bryant, 2006).

### 2.3 Demographic analysis

**Figure 1:**
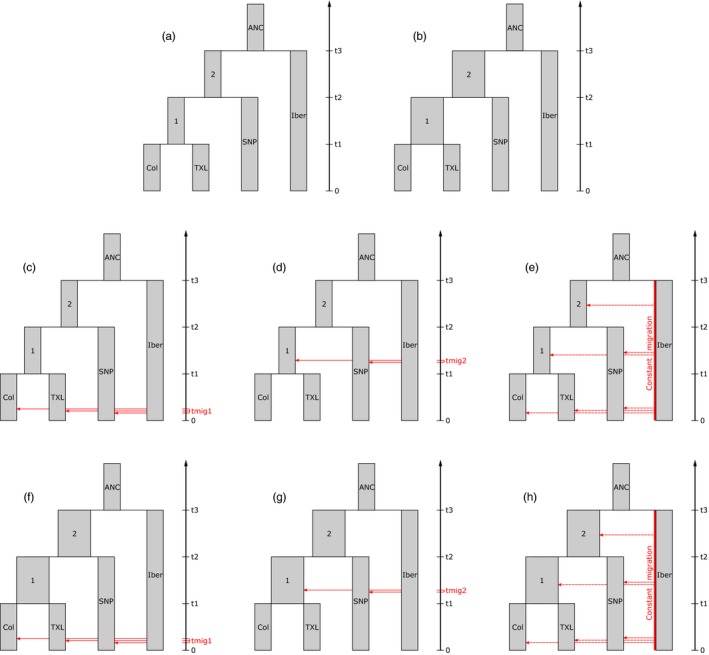
Modelled scenarios for reconstructing Creole cattle demographic history using approximate Bayesian computation (ABC). (a) Scenario 1: main model of cattle dispersion throughout the Americas. (b) Scenario 2: variation that includes expansions in Creole populations at t2 and t3. (c) Scenario 3: variation that includes recent migration. (d) Scenario 4: variation that includes migration before t1. (e) Scenario 5: variation that includes ongoing migration. (f) Scenario 6: variation that combines scenarios 2 and 3. (g) Scenario 7: variation that combines scenarios 2 and 4. (h) Scenario 8: variation that combines scenarios 2 and 5

The population history of Creole cattle was reconstructed from the late 15th century to the present day using approximate Bayesian computation (ABC) as in Pitt et al. (2018). Briefly, a subset of the data was divided into four clusters: Col including all Colombian breeds (Costeño con Cuernos, Romosinuano, San Martinero), Senepol, Texas Longhorn and Iber (for all Iberian breeds). Eight alternative demographic histories were modelled based on historical records, results from Admixture, MDS and neighbour‐net analyses, and prior *N*_e_ estimates obtained with SNeP. The scenarios included a model of Creole cattle dispersal throughout the Americas and variations of this model accounting for population expansions and alternative migration patterns representing restocking from Iberian populations (Figure 1). One million reverse coalescent simulations were generated for each of the eight scenarios with Fastsimcoal2 (Excoffier, Dupanloup, Huerta‐Sánchez, Sousa, & Foll, 2013; Excoffier & Foll, 2011) using a pipeline implemented in ABCtoolbox (Wegmann et al., 2010), with a required computation time of eight days per scenario splitting simulations in ~50 parallel runs. Seventeen summary statistics were calculated in Arlsumstat (Excoffier & Lischer, 2010) for simulated and observed data (Supporting Information Table S2). A Spearman’s rank correlation was calculated between each pair of summary statistics in R, and statistics with consistently high negative or positive correlation were removed (Supporting Information Figure S1, Table S2). ABCtoolbox was used to perform rejection sampling on the simulated data set, retaining the 5,000 (0.5%) simulations that closest fit to the observed data for each of the eight scenarios. Marginal density (MD) and posterior probability *P*‐values (i.e., the proportion of simulations that have a smaller or equal likelihood to the observed data) were calculated from the retained simulations after a postsampling regression adjustment using a general linear model. Bayes factors (BF) were calculated between scenarios by taking the quotient of the MD from two scenarios to choose the best modelled scenario fitting our data (i.e., if BF > 3, the alternative scenario can be rejected—Wegmann et al., 2010‐).

To examine the most recent changes in *N*_e_, the software SNeP v1.11 (Barbato et al., 2015) was used to estimate the demographic history for each population by the relationship between LD and *N*_e_ up until approximately 13 generations in the past. Default options were used apart from sample size correction for unphased genotypes, correction to account for mutation and Sved and Feldman’s (1973) mutation rate modifier. To identify subtle changes in the inferred *N*_e_ curve that might be diagnostic of changes in *N*_e_ not visually explicit when observed in the *N*_e_ plot, a “*N*_e_ Slope analysis” (NeS) was used to investigate the rate and directionality of *N*_e_ changes occurring in recent generations (Supporting Information Figure S2). The slope of each segment linking pairs of neighbouring *N*_e_ estimates was first calculated and then normalized using the median of the two most proximal past *N*_e_ slope values as in ${\text{NeS}}_{n}=\left(S_{n}-{\tilde{X}}_{n}\right){\left(1+{\tilde{X}}_{n}\right)}^{-1}$ where *S*_*n*_ is the slope of the *n* ^th^ pair of neighbouring *N*_e_ estimates, and ${\tilde{X}}_{n}\;=\;\text{med}\left\{S_{n},S_{n\;+\;1},S_{n\;+\;2}\right\}$.

### 2.4 Selection signatures

We scanned for recently generated selection signatures to characterize differences observed between breeds that have remained in the Iberian Peninsula and those that colonized the Americas. Four Creole clusters were selected using the Admixture, MDS and neighbour‐net results, one group (Col) including the three Colombian breeds (Costeño con Cuernos, Romosinuano, San Martinero) and three other breeds from the Americas, Florida Cracker, Senepol and Texas Longhorn. All pairwise comparisons were analysed between these four Creole clusters and three Iberian clusters used as biological replicates: (i) IB1, including Retinta, Berrenda en Colorado and Cachena; (ii) IB2, including Cárdena Andaluza, Asturiana de los Valles, Pajuna and Mostrenca; and (iii) a third group (LID), including the six Lidia lineages. The data set was separated per breed using VCFtools 0.1.15 (Danecek et al., 2011), and haplotype reconstruction was carried out using Beagle. All missing data were removed from the merged data set of the four groups using VCFtools and leaving 15,375 SNPs.

Recent selective sweeps were identified in the Creole populations with the software Selscan 1.1.0b (Szpiech & Hernandez, 2014) using XP‐EHH (Sabeti et al., 2007) with the IB1, IB2 and LID groups as references. The maximum distance between adjacent SNPs was 500 kb to allow for inconsistencies in bovine SNP arrays, whereas the remainder of the settings were left as default. The XP‐EHH scores were standardized across the whole genome. XPEHH scores exceeding the extreme 1% of the standardized distribution were identified as potential locations for positive selection in each given Creole cluster. All significant SNPs of a Creole breed validated with at least two Iberian clusters were merged regardless of the Iberian ancestral group to account for breed specific selection signatures. Contiguous significant SNPs were integrated to a common signature or region within each breed, allowing for one nonsignificant SNP in the middle, and including half of the physical distance to the neighbouring nonsignificant marker on both sides. As XP‐EHH searches for unusually long haplotypes, isolated significant SNPs were discarded, rendering this analysis conservative.

Selection signatures expected to have been generated prior to colonization of the Americas were explored using *F*_ST_ outliers compared to the null distribution generated in nonoverlapping windows of 500 kb using VCFtools. We used a windowed *F*_ST_ as a test statistic, retaining windows with values exceeding the 99% upper quantile as potential locations for selection. Given that *F*_ST_ analysis is not directional, that is does not differentiate between Creole or Iberian signatures of selection, only windows validated in the three Iberian replicates were consider for downstream analysis to isolate signals detected only in Creole cattle.

### 2.5 Ancestry estimation at candidate regions

Local Ancestry in adMixed Populations (LAMP) version 2.5 (Pasaniuc, Sankararaman, Kimmel, & Halperin, 2009) was used to estimate the ancestry proportions (Iberia, commercial, Africa and zebu) of Creole breeds at candidate regions. We applied the LAMPANC method for inferring the locus‐specific ancestries providing the genotypes of the ancestral populations. Autosome‐wide Creole ancestry proportions of 76% Iberian, 12% commercial and 3% African taurine groups, and 9% zebu cattle were estimated from the Admixture proportions *α*. An estimated number of 83 generations was set for the beginning of admixture in Creole cattle taking into account the introductions of North‐European cattle in North America between 1608 and 1640 (Felius et al., 2014), assuming an average generation length of 5 years, and otherwise using default parameters. The average excess/deficiency in the different ancestries was calculated by subtracting the average estimated ancestry at each significant SNP within candidate regions from the average estimated ancestry of all SNPs.

### 2.6 Gene ontology analysis

Gene ontology (GO) analysis was carried out on the annotated gene sets included in genomic regions under selection in Colombian, Florida Cracker, Senepol and Texas Longhorn breeds using the Functional Annotation Cluster (FAC) tool from the Database for Annotation, Visualization and Integrated Discovery (DAVID) v6.8 (Huang, Sherman, & Lempicki, 2009) to determine significantly enriched biological functions or processes positively selected in a breed using high stringency ease scores. KEGG pathway analyses were also performed in DAVID to map clusters of genes involved in common pathways. In addition, the Bovine QTL Animal database (http://www.animalgenome.org) was used to identify any overlap with quantitative trait loci (QTL) described in the literature.

## 3 RESULTS AND DISCUSSION

Tropical adaptation, that is the ability to tolerate heat stress, high humidity, tropical diseases and parasite infections while maintaining standards of performance and reproduction, constitutes the most valuable asset of Creole cattle, assuring protein production within its region and providing insights into genomic and physiologic mechanisms selected during the transition to a tropical environment. Most Creole breeds included in this study (Costeño con Cuernos, Romosinuano, San Martinero, Florida Cracker, Senepol) have been developed under physiologically challenging tropical conditions and tolerate high temperatures and humidity, poor soils, drought, high rainfall, and are tick resistant, all while maintaining good performance (de Alba, 1987). In addition, breeds such as Texas Longhorn have adapted to very hot and dry tropical conditions including the ability to reproduce very effectively with minimal human intervention where forage is sparse.

### 3.1 Autosomal ancestry proportions and population divergence in Creole cattle breeds

**Figure 2:**
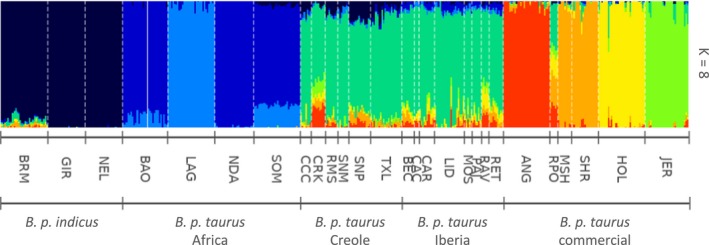
Ancestry proportions in Creole breeds at *K* = 8. Complete breed names are included in Supporting Information Table S1

Admixture analysis when the number of clusters was set to eight, depicting zebu, African (two clusters), Iberian, Angus, Shorthorn, Holstein and Jersey ancestry contributions to Creole populations ascribed the major genomic component to Iberian ancestry (0.76 ± 0.06 *SD*), with minor influences from zebu and European commercial breeds (Table 1, Figure 2), in concordance with previous studies (Decker et al., 2014; Martínez et al., 2012). Among Creole breeds, the Florida Cracker displayed the highest level of introgression from commercial genomes (0.36 ± 0.07 *SD*), mainly from Jersey, Angus and Shorthorn, whereas the Indicine component was higher in Senepol (0.15 ± 0.02 *SD*) and Romosinuano (0.10 ± 0.02 *SD*). Creole populations included in this study were largely unaffected by the introduction of African taurine cattle into the Americas, which reached its highest proportion in the San Martinero and Texas Longhorn (0.05 ± 0.01 *SD* ; Table 1, Figure 2). This residual African genomic component may be explained by ancient introgression in the Iberian Peninsula and the Canary Islands (McTavish, Decker, Schnabel, Taylor, & Hillis, 2013).

**Table 1:** Average taurine and indicine ancestries in Creole cattle breeds

Breed	*B. p. taurus* Iberia	*B. p. taurus* commercial	*B. p. taurus* Africa	*B. p. indicus*
	Mean ± *SD*	Mean ± *SD*	Mean ± *SD*	Mean ± *SD*
Costeño con Cuernos	0.80 ± 0.08	0.07 ± 0.06	0.04 ± 0.01	0.09 ± 0.03
Florida Cracker	0.60 ± 0.03	0.36 ± 0.07	0.01 ± 0.01	0.03 ± 0.02
Romosinuano	0.80 ± 0.06	0.07 ± 0.04	0.03 ± 0.01	0.10 ± 0.02
San Martinero	0.86 ± 0.06	0.04 ± 0.03	0.05 ± 0.01	0.06 ± 0.04
Senepol	0.69 ± 0.05	0.14 ± 0.04	0.02 ± 0.01	0.15 ± 0.02
Texas Longhorn	0.81 ± 0.07	0.06 ± 0.03	0.05 ± 0.01	0.08 ± 0.06
Mean	0.76 ± 0.06	0.12 ± 0.05	0.03 ± 0.01	0.09 ± 0.03

**Figure 3:**
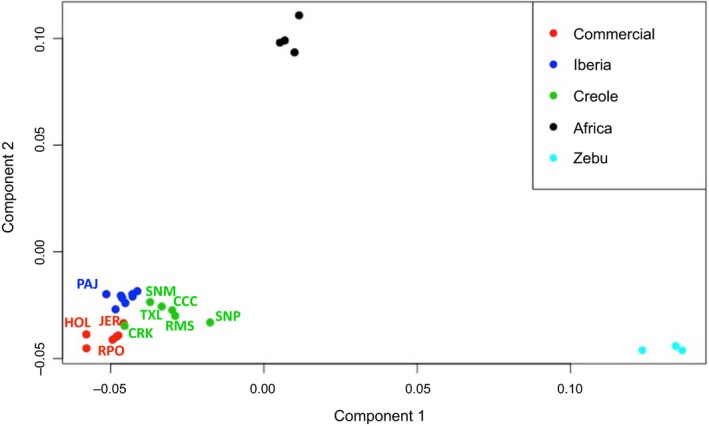
Multidimensional scaling (MDS) plot for 27 taurine and indicine cattle populations

**Figure 4:**
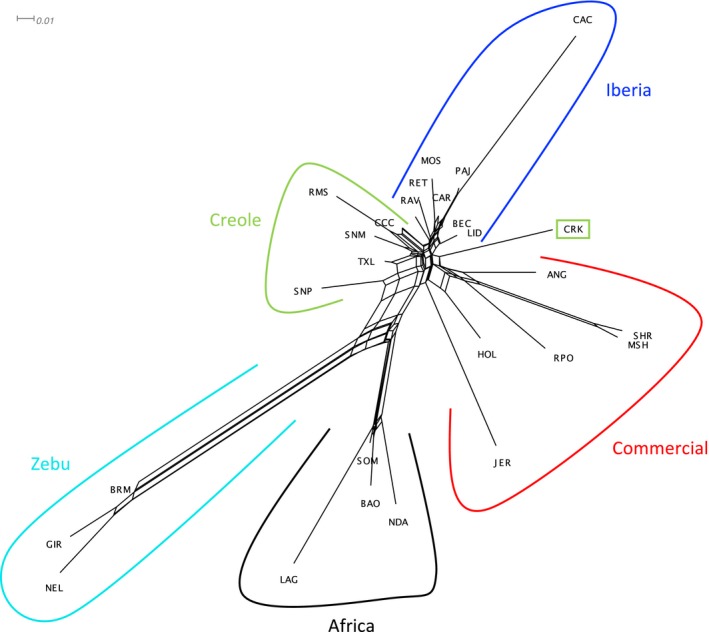
Neighbour‐net using Reynolds’ distances for 27 taurine and indicine cattle populations. Scale for Reynolds’ distance is displayed in the top left

Multidimensional scaling allocated ~25% and ~21% of the variance to the first two axes, respectively, which separated taurine from zebu cattle breeds, and African taurine from the remaining populations (Figure 3). Among the relationships displayed by Creole, Iberian and commercial breeds, Senepol showed the highest differentiation, driven by the influence of zebu breeds, and Florida Cracker was grouped most closely with the commercial breeds. These results were supported by the neighbour‐net analysis, which clustered the breeds into five main groups (zebu, Africa, commercial, Iberia and Creole), with Florida Cracker intermediate between Iberian and the commercial breeds (Figure 4).

The high contributions of zebu (15%) and European commercial breeds (14%) with minor elements of African taurine ancestry (2%) found in Senepol are in accordance with the results obtained by Flori et al. (2012) and Huson et al. (2014), and they argue against the reporting of direct incorporation of N’Dama into Senepol breeding (Miretti et al., 2004). Although these authors attributed all European taurine contribution to Red Poll ancestry, our results strongly imply a major Iberian origin (68%) with a much lower ancestral contribution from commercial breeds (14%), including Red Poll. Despite the claimed admixture of Romosinuano with polled British breeds to incorporate polledness into its phenotype (Huson et al., 2014), contribution from European commercial breeds (including Red Poll samples) was inferred to be low and equal to that of Costeño con Cuernos (7%), from which the Romosinuano was developed. Although theoretically Florida Cracker has not been crossed with European commercial breeds (Ekarius, 2008), this ancestry represents 36% of its genomic pool. Finally, despite indicine introgression having been described in the Texas Longhorn (Decker et al., 2014), the values detected here are within the mean range for all Creole cattle populations (8%).

These results illustrate the influence of taurine and indicine ancestry that may underlie some of the demographic patterns and selection signatures found in Creole populations.

### 3.2 Demographic history

ABC modelling was used to explore the recent demographic history of Creole cattle from the arrival of the first individuals to the Americas at the end of the 15th century to present. Thirteen summary statistics were retained after removing correlated measurements (Supporting Information Figure S1, Table S2). All observed summary statistics were within the 95% quantiles of the simulated summary statistics for each scenario. Comparison of the different scenarios showed a BF > 3 between scenarios 2, 6 and 7 and all the others (Table 2). Among the three best fitting scenarios, scenario 2 displayed the highest MD value, with a BF of 1.4 and 1.7 when compared with scenarios 6 and 7, respectively (Table 2). Scenario 2 supports the participation of a small number of animals (84) in the development of American breeds, followed by a major expansion up to a *N*_e_ of 57,278 180 YA, that later on collapsed to the reduced population sizes detected nowadays, ranging between 497 for Senepol and 638 for Texas Longhorn (Table 3, Supporting Information Figure S3). Higher *N*_e_ values were retrieved for the Colombian (755) and Iberian (2,577) breeds derived from the grouping of three and eight populations, respectively, which overestimated diversity values and therefore provide a rough estimation of effective population sizes of around 252 (Colombia) and 322 (Iberia) genomes per breed in each group. These events are in close agreement with the known history of foundation, expansion and later contraction of cattle of Iberian origin in the Americas (de Alba, 1987; Eusebi, Cortés, Dunner, & Cañón, 2017; Rodero et al., 1992; Villalobos Cortés et al., 2009; Willham, 1982), and with the general trend displayed by populations that successfully colonize new habitats, undergoing a bottleneck followed by rapid growth usually due to lack of competition but here more likely due to habitat modification (see Gray et al., 2014 for a review).

**Table 2:** Approximate Bayesian computation (ABC) results for the different scenarios (shown in Figure 1) modelling Creole cattle demographic history

Scenario	*P*‐value	Marginal density	Bayes factor							
			Sc. 1	Sc. 2	Sc. 3	Sc. 4	Sc. 5	Sc. 6	Sc. 7	Sc. 8
Sc. 1	0.42	308.1	—	0.05	0.66	0.75	3.31	0.08	0.09	2.16
Sc. 2	0.67	5627.8	18.27	—	12.06	13.75	60.45	1.41	1.69	39.38
Sc. 3	0.56	466.5	1.51	0.08	—	1.14	5.01	0.12	0.14	3.26
Sc. 4	0.38	409.2	1.33	0.07	0.88	—	4.40	0.10	0.12	2.86
Sc. 5	0.52	93.1	0.30	0.02	0.20	0.23	—	0.02	0.03	0.65
Sc. 6	0.82	3993.2	12.96	0.71	8.56	9.76	42.89	—	1.20	27.94
Sc. 7	0.69	3324.4	10.79	0.59	7.13	8.12	35.71	0.83	—	23.26
Sc. 8	0.42	142.9	0.46	0.03	0.31	0.35	1.53	0.04	0.04	—

**Table 3:** Prior distributions and posterior characteristics for scenario 2, the preferential ABC model with and expanded Creole population between t3 and t1

Parameter	Prior distributions^a^			Posterior characteristics				
	Scale	Minimum	Maximum	Mode	Q50 lower	Q50 upper	Q90 lower	Q90 upper
Mutation rate	Log_10_	0.0001	0.05	0.00214	0.00185	0.00292	0.00143	0.00413
*N*_e__1	Log_10_	100	500,000	57,278	10,936	116,464	2,015	343,384
*N*_e__2	Log_10_	100	500,000	40,765	8,262	99,131	1,467	32,5147
*N*_e__ANC	Log_10_	100	5,000	84	61	111	39	167
*N*_e__Iber	Log_10_	100	50,000	2,577	1,725	3,975	949	7,236
*N*_e__TXL	Log_10_	10	5,000	638	376	1,157	176	2,515
*N*_e__Col	Log_10_	10	50,000	755	378	1,622	137	4,676
*N*_e__SNP	Log_10_	10	5,000	497	356	694	224	1,094
t1^b^	Linear	5	150	36	28	68	11	100
t2^b^	Linear	20	150	89	64	110	36	136
t3^b^	Linear	50	150	127	92	130	64	145

Log_10_ scaled priors have been converted back from Log_10_.

Q50, 50th quantile range; Q90, 90th quantile range; *N*_e__1, effective population size at t1; *N*_e__t2, effective population size at t2; *N*_e__ANC, ancestral effective population size; *N*_e__Iber, Iberian cluster effective population size; *N*_e__TXL, Texas Longhorn effective population size; *N*_e__Col, Colombian cluster effective population size; *N*_e__SNP, Senepol effective population size.

a Priors were sampled uniformly.

b Time in generations, assuming a generation length of 5 years.

To build realistic models, we used ABC analysis with priors guided by historical population and migration records, Admixture, MDS and neighbour‐net results, and recent *N*_e_ estimations based on LD, and included a wide representation of the Iberian populations sharing a common ancestor with Creole breeds in the recent past. However, obtaining exact parameter estimates can be complex (Gray et al., 2014), which may explain the discrepancy we found between the colonization time t3 (635 YA) and known dates such as the arrival of cattle to the Americas after 1492 (524 YA; although within the 50th quartile range of 460–650 years). However, the drastic *N*_e_ reduction from t1 (180 YA) to present closely correlates with the introduction of zebu and commercial cattle breeds to the Americas, starting around the middle of the 19th century and causing the gradual replacement of Creole populations that has led to their small current effective population sizes (de Alba, 1987; Willham, 1982). Despite the influence of European commercial breeds and zebu cattle detected here and supported by historical records (Decker et al., 2014; Felius et al., 2014), computational constraints hampered their incorporation in the models. It is possible that the potential oversimplification of the models analysed here may underestimate the complex demography of Creole breeds and obscure recent Iberian, European and zebu influences.

**Figure 5:**
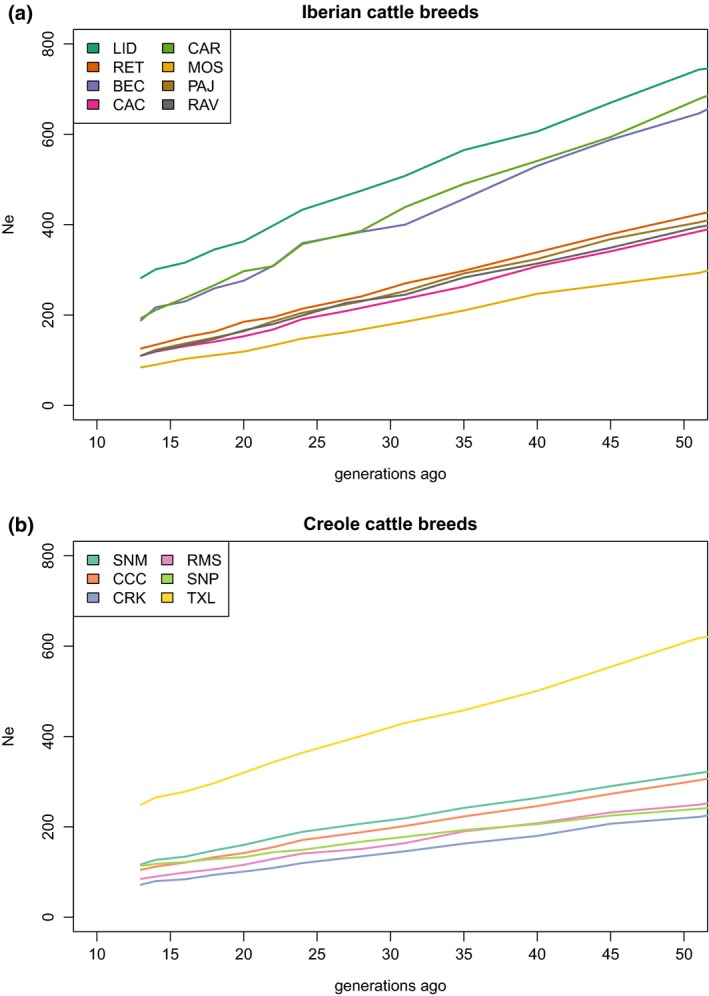
Estimation of *N*_e_ change between 13 and 50 generations ago using SNeP

The LD approach implemented in the SNeP program recorded a declining trend in *N*_e_ for all cattle breeds since 250 YA (Figure 5), also captured by the ABC analysis, which is likely to reflect reductions in gene flow between herds and the start of breed formation (MacLeod, Larkin, Lewin, Hayes, & Goddard, 2013; Taberlet et al., 2011), as well as the replacement of Creole populations. The Iberian populations converged in three distinct clusters, one including Berrenda en Colorado, Lidia and Cárdena Andaluza, with a second including Cachena, Asturiana de los Valles, Retinta and Pajuna, and a third including Mostrenca (Figure 5a). These distinct demographic trajectories may correspond to relatively ancient branches such as Black Iberian for Lidia and Cárdena Andaluza, Cantabrian for Cachena and Asturiana de los Valles, or the individual trajectory of Mostrenca, a very ancient semi‐feral breed uniquely adapted to the seasonally inundated marshes of Las Marismas in Andalucia (MARM, 2010). Creole breeds produced more homogeneous demographic trajectories, apart from the Texas Longhorn (Figure 5b).

**Figure 6:**
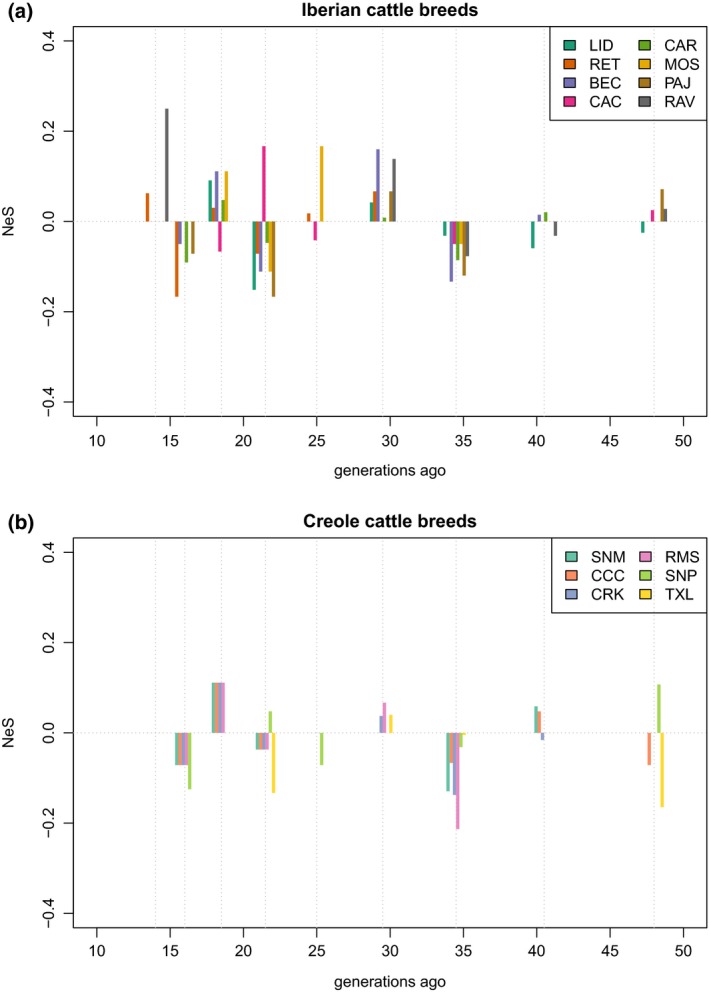
*N*_e_ Slope analysis (NeS) between 13 and 50 generations ago

To further investigate the complex, recent demographic trajectories NeS was used. The novel NeS method records the change in slope of the inferred *N*_e_ trend obtained from LD‐based demography analysis implemented in SNeP, potentially offering a more detailed picture of population changes 13–50 generations ago; a constant rate of change is shown as a flat line proximal to 0 in the *Y*‐axis, whereas deviations above and below 0 represent relative increases and reductions in *N*_e_, respectively (Supporting Information Figure S2). This analysis depicted a decrease in *N*_e_ towards the end of the expansion period, followed by a temporary recovery in effective size before a collapse to the small *N*_e_ detected in the present day (Figure 6). Thus, after several recent fluctuations, the current very small *N*_e_ was attained only towards the end of the 20th century. The majority of the Iberian breeds recorded similar overlapping NeS patterns (Figure 6a). A slowly increasing reduction in *N*_e_ being recorded until ~35 generations ago, followed by several fluctuations in *N*_e_, until ~16 generation in the past where a marked reduction in *N*_e_ is shown. Among the breeds, the Cachena showed the opposite pattern between ~22 and ~18 generations ago, depicting a sharp increase followed by a reduction in *N*_e_. In contrast (also with all other breeds), Mostrenca expanded ~25 generation in the past, as well as Asturiana de los Valles in recent generations (~15). The majority of Creole breeds recorded overlapping NeS patterns (Figure 6b) and mirrored those recorded by the Iberian breeds, with Senepol displaying a different pattern until very recently (~18 generations ago), whereupon it converges with the other breeds showing an increase followed by a steep population decline.

The difference in inference gained using ABC and SNeP is likely to reflect their resolution of temporal complexity, where ABC only allows comparison among competing demographic scenarios whereas SNeP applies a single, model‐free algorithm and its application enables the inference of more complex, short‐term, events instead. Thus, ABC reveals general trends and their relative likelihood, while LD‐based analysis provides an insight on the short‐term complexity within these trends.

### 3.3 Signatures of selection

We applied two methodologies that analyse different patterns of genetic variation, mainly related to evolutionary timescale, to investigate selection pressures enforced by the new tropical environment in six Creole populations, five of which are adapted to humid and hot conditions and one to dry and hot conditions. We used *F*_ST_, better suited to detect signals in the more distant past (Sabeti et al., 2006) that might reflect the zebu ancestral component found in Creole populations, and the LD‐based XP‐EHH method, which provides better resolution for recent selection (Cadzow et al., 2014) and is more suitable for disentangling the differences between Creole and Iberian populations expanding over the last 500 years.

**Figure 7:**
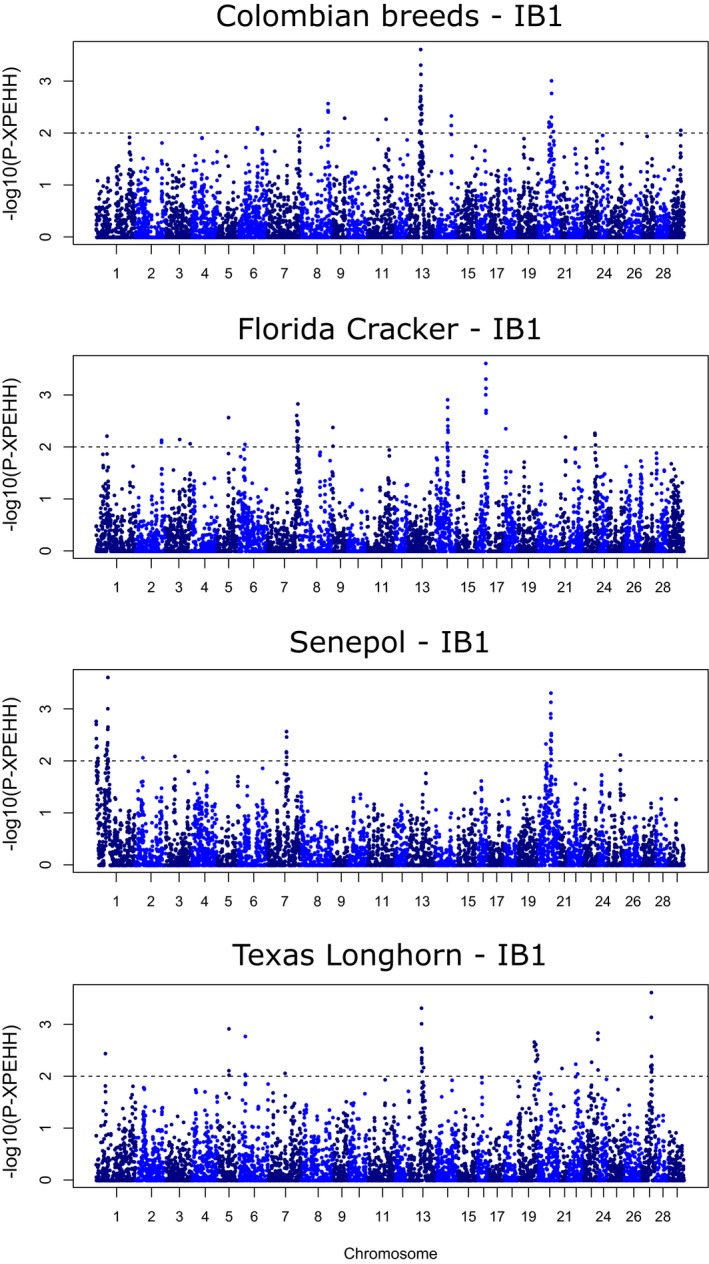
Manhattan plots of genomewide distribution of selection signatures detected with XP‐EHH for Creole clusters when compared to the Iberian ancestral group IB1. Threshold is set at −log_10_(P‐XPEHH) = 2

Figure 7 and Supporting Information Figures S4–S5 depict the genomewide distribution of outliers on each autosome detected by XP‐EHH and *F*_ST_ scans for signatures of selection. The total number of significant SNPs and windows identified per cluster is listed in Supporting Information Tables S3 and S4. Using the criteria of contiguous blocks of at least two SNPs from the XP‐EHH analysis confirmed with more than one Iberian group, or windows containing two or more SNPs from the *F*_ST_ analysis confirmed with the three Iberian groups, we retrieved 10–14 genomic regions under selection per Creole cluster—two shared between Colombian and Texas Longhorn breeds, one between Colombian and Senepol clusters, and one between Florida Cracker and Texas Longhorn—(Table 4). Annotation of genomic regions under selection from both analyses retrieved 38, 66, 72 and 61 different genes in the Colombian, Florida Cracker, Senepol and Texas Longhorn clusters, respectively (Table 4). GO analysis using DAVID produced a total of 12 enriched functional clusters (Supporting Information Table S5) and 13 enriched KEGG signalling pathways (Table 5).

**Table 4:** Genomic regions under positive selection detected with *F*_ST_ and XP‐EHH analyses in Creole breeds

Region	SNPs	Method	BTA position	Region length (kbp)	∆AI^a^	∆AC^a^	∆AA^a^	∆AZ^a^	Candidate genes	QTL
					Whole genome ancestry (mean ± *SD*)^b^					
Colombian breeds (Col)					0.90 ± 0.08	0.03 ± 0.04	0.01 ± 0.02	0.06 ± 0.06		
#1	2	XP‐EHH	1:96530234‐97142235	612	0.08	−0.03	−0.01	−0.04	*PLD1, TNIK, ENSBTAG00000031795*	—
#2	2	*F*_ST_	3:83000001‐83500000	500	0.03	−0.01	−0.01	−0.01	*ATG4C, U6, ENSBTAG00000048179*	Milk, reproduction
#3	3	*F*_ST_	5:19500001‐20000000	500	0.07	−0.03	0.01	−0.05	*ATP2B1, 5S_rRNA*	Tick resistance, weight, performance, milk
#4	3	XP‐EHH	6:95081924‐95220410	138.5	−0.01	0.04	−0.01	−0.01	*ANXA3*	Milk
#5	3	XP‐EHH	6:115330158‐115581341	251.2	−0.04	0.02	−0.01	0.03	*C1QTNF7, CC2D2A, bta‐mir‐2448, FBXL5*	—
#6	3	XP‐EHH	8:104528330‐104765557	237.2	−0.01	−0.03	0.04	0.01	*RGS3, ENSBTAT00000011467*	—
#7	2	XP‐EHH	13:38621621‐38870180	248.6	−0.07	−0.01	0.01	0.07	*KAT14, ENSBTAG00000004620, ENSBTAG00000004620, DZANK1, POLR3F, RBBP9*	Weight
#8	5	XP‐EHH	13:39065177‐39654999	589.8	−0.08	−0.01	0.01	0.08	*SLC24A3*	Milk, reproduction, feed intake
#9	11	XP‐EHH	13:39880764‐40951781	1071	−0.08	−0.03	0.01	0.10	*NAA20, CRNKL1, CFAP61, INSM1, RALGAPA2, SNORA70, KIZ*	Feed intake, conformation, weight, reproduction, milk
#10	3	XP‐EHH	13:41726060‐42207435	481.4	−0.06	−0.03	0.01	0.08	*FOXA2, U6*	Conformation, weight, reproduction
#11	9	XP‐EHH	20:35850633‐37219008	1368.4	**−0.39**	−0.03	0.04	**0.38**	*LIFR, EGFLAM, SNORA17, U6, GDNF, WDR70, NUP155, bta‐mir‐2360, ENSBTAG00000000586, NIPBL*	Slick hair coat, milk, mastitis, feed intake, meat, reproduction, weight
Florida Cracker (CRK)					0.57 ± 0.16	0.27 ± 0.13	0.03 ± 0.07	0.13 ± 0.13		
#12	2	XP‐EHH	5:44487133‐44773477	286.3	−0.07	−0.05	0.03	0.09	*ENSBTAG00000039170, ENSBTAG00000026323, ENSBTAG00000026088, ENSBTAG00000020564, ENSBTAG00000046511, ENSBTAG00000046628, ENSBTAG00000026322, U6, LYZ, CPSF6*	Reproduction (tropical breed)
#13	3	XP‐EHH	6:12946249‐13195583	249.3	−0.18	−0.10	0.08	0.20	*CAMK2D*	Weight, milk
#14	2	XP‐EHH	7:97937246‐98208707	271.5	−0.18	−0.16	−0.03	**0.37**	*PCSK1*	Weight, conformation, meat
#15	4	XP‐EHH	7:98584703‐98897359	312.7	−0.18	−0.16	−0.03	**0.37**	*ERAP1, ERAP2, LNPEP*	Reproduction, weight, performance
#16	6	XP‐EHH	7:107116333‐107853496	737.2	−0.24	−0.16	−0.03	**0.43**	*ENSBTAG00000000360*	Meat
#17	2	XP‐EHH	9:8595005‐9165093	570.1	**−0.35**	0.06	−0.03	**0.31**	*LMBRD1*	Milk
#18	2	*F*_ST_	11:105500001‐106000000	500	−0.13	0.12	0.03	−0.02	*ZMYND19, ARRDC1, DPH7, PNPLA7, MRPL41, NSMF, NOXA1, ENTPD8, EXD3, NRARP, TOR4A, FAM166A, NDOR1, CYSRT1, RNF224, NELFB, SSNA1, TUBB4B, LRRC26, SLC34A3, RNF208, ENSBTAG00000046416, ENSBTAG00000046223, TMEM203, ENSBTAG00000047715, TMEM210, GRIN1, RXRA*	Milk, reproduction, conformation, fatty acids
#19	15	XP‐EHH	14:25682788‐26937892	1255.1	−0.13	−0.10	0.03	0.20	*FAM110B, ENSBTAG00000047136, UBXN2B, CYP7A1, U1, SDCBP, NSMAF, TOX*	Tick resistance, reproduction, insulin growth factor, milk, weight
#20	6	XP‐EHH	16:76321854‐77156723	834.9	**−0.51**	0.17	0.03	**0.31**	*SNORA48, PLXNA2*	Weight, reproduction, daily gain, performance, conformation, milk
#21	2	*F*_ST_	21:29500001‐30000000	500	**0.32**	−0.21	−0.03	−0.07	*PCSK6, SNRPA1, ENSBTAG00000003957, ENSBTAG00000047130*	Reproduction, performance
#22	3	XP‐EHH	23:45450666‐45712365	261.7	0.15	−0.05	−0.03	−0.07	*TFAP2A*	Tuberculosis susceptibility, weight, milk
#23	3	XP‐EHH	23:46206000‐46470169	264.2	0.15	−0.05	−0.03	−0.07	*—*	—
#24	4	*F*_ST_	26:37500001‐38000000	500	−0.24	−0.16	−0.03	**0.43**	*ENO, SHTN1, VAX1, KCNK18, SLC18A2, PDZD8*	Heat tolerance, temperament, reproduction, milk
Senepol (SNP)					0.64 ± 0.15	0.12 ± 0.10	0.02 ± 0.04	0.21 ± 0.13		
#25	6	XP‐EHH, *F*_ST_	1:1000001‐1908934	908.9	**−0.35**	−0.08	−0.02	**0.47**	*ITSN1, CRYZL1, DONSON, SON, GART, DNAJC28, TMEM50B, SNORA20, IFNGR2, IFNAR1, ENSBTAG00000019404, IFNAR2, HIST1H4G, OLIG1*	Polled, milk, reproduction
#26	7	XP‐EHH, *F*_ST_	1:2188833‐3000000	811.2	**−0.35**	−0.08	−0.02	**0.47**	*EVA1C, URB1 (splice region variant), MRAP, MIS18A, HUNK*	Weight, performance, reproduction, milk, conformation, stature
#27	2	XP‐EHH	1:6557886‐7047431	489.5	**−0.35**	−0.08	−0.02	**0.47**	*LTN1, ENSBTAG00000038433, N6AMT1, U6*	—
#28	3	XP‐EHH	1:63614355‐64126677	512.3	−0.21	−0.12	−0.02	**0.36**	*—*	Milk, meat
#29	9	XP‐EHH	1:64565646‐65264693	699	−0.21	−0.12	−0.02	**0.36**	*UPK1B, B4GALT4, ARHGAP31, TMEM39A, POGLUT1, TIMMDC1, CD80, ADPRH, PLA1A, POPDC2, COX17, MAATS1, SNORA31, NR1I2*	Meat, milk, reproduction
#30	2	*F*_ST_	5:90000001‐90500000	500	−0.14	0.09	0.02	0.04	*ENSBTAG00000044467, 5S_rRNA*	Reproduction, milk, conformation, stature
#31	5	XP‐EHH	7:64259215‐65018918	759.7	0.07	−0.12	−0.02	0.08	*GPX3, TNIP1, ANXA6, CCDC69, ENSBTAG00000045615, GM2A, SLC36A3, SLC36A2, SLC36A1, ENSBTAG00000003498, ENSBTAG00000047625, SPARC, ATOX1, G3BP1*	Milk, reproduction, conformation, performance,
#32	2	*F*_ST_	12:32000001‐32500000	500	0.11	−0.08	−0.02	0.00	*FLT3, URAD, ENSBTAG00000001819, PDX1, ENSBTAG00000009166*	Weight
#33	14	XP‐EHH	20:35850633‐38012333	2161.7	**−0.37**	−0.08	0.02	**0.45**	*LIFR, EGFLAM, SNORA17, U6, GDNF, WDR70, NUP155, bta‐mir‐2360, ENSBTAG00000000586, NIPBL, ENSBTAG00000047208, SLC1A3, RANBP3L*	Slick hair coat, milk, mastitis, feed intake, meat, reproduction, weight
#34	2	*F*_ST_	21:16500001‐17000000	500	0.04	−0.05	−0.02	0.04	*U6, ENSBTAG00000037383, KLHL25*	Reproduction, milk, tuberculosis susceptibility
Texas Longhorn (TXL)					0.87 ± 0.07	0.04 ± 0.04	0.02 ± 0.03	0.07 ± 0.05		
#35	2	XP‐EHH	1:147742602‐147862779	120.2	0.01	0.01	0.03	−0.05	*PCNT, DIP2A*	Weight
#36	3	*F*_ST_	2:33500001‐34000000	500	0.01	−0.02	−0.02	0.03	*ENSBTAG00000047523*	Fat, conformation, reproduction, performance, stature
#37	3	XP‐EHH	2:71689118‐72269356	580.2	0.01	0.04	−0.02	−0.02	*SCTR, CFAP221, TMEM177, PTPN4, ENSBTAG00000048209, EPB41L5*	—
#38	4	XP‐EHH	5:44373006‐45012918	639.9	**−0.17**	−0.02	**0.06**	**0.13**	*U5, ENSBTAG00000022971, ENSBTAG00000000198, ENSBTAG00000039170, ENSBTAG00000026323, ENSBTAG00000026088, ENSBTAG00000020564, ENSBTAG00000046511, ENSBTAG00000046628, ENSBTAG00000026322, U6, LYZ, CPSF6, ENSBTAG00000002741, SNORA44*	Reproduction (tropical breed), meat, milk
#40	3	XP‐EHH	6:19530474‐20352600	822.1	0.00	−0.02	0.01	0.00	*GIMD1, AIMP1, TBCK, ENSBTAG00000043620*	Milk, meat, weight
#41	5	XP‐EHH	6:115330158‐115857381	527.2	0.01	−0.04	−0.02	0.06	*C1QTNF7, CC2D2A, bta‐mir‐2448, FBXL5, U6, BST1, CD38*	‐
#42	10	XP‐EHH	13:39981697‐40951781	970.1	−0.12	−0.04	**0.16**	0.00	*CFAP61, INSM1, RALGAPA2, SNORA70, KIZ*	Feed intake, conformation, weight, reproduction, milk
#43	4	XP‐EHH	13:46248573‐46574633	326.1	0.08	−0.04	0.01	−0.05	*ADARB2, ENSBTAG00000039356, ENSBTAG00000037833*	Milk
#44	3	XP‐EHH	14:7907751‐8007829	100.1	−0.05	−0.02	0.01	0.06	*—*	Milk
#45	3	XP‐EHH	15:59919746‐60219850	300.1	−0.05	−0.02	0.03	0.03	*—*	Reproduction, conformation, performance
#46	3	XP‐EHH	23:51003189‐51242238	239	0.01	−0.02	0.01	0.00	*ENSBTAG00000037624, ENSBTAG00000012058*	Meat, milk, reproduction
#47	3	XP‐EHH	24:57909464‐58455030	545.6	0.08	−0.04	0.01	−0.05	*NEDD4L, bta‐mir‐122, ALPK2, MALT1*	Abomasum displacement, milk, meat
#48	4	XP‐EHH	27:35316858‐36020332	703.5	0.11	−0.04	0.01	−0.07	*ZMAT4, 5S, SNORA70, SFRP1*	Milk
#49	6	XP‐EHH	27:36220416‐36884199	663.8	0.11	−0.04	0.01	−0.07	*GPAT4, ENSBTAG00000047361, ENSBTAG00000004244, bta‐mir‐486, ENSBTAG00000004242, ENSBTAG00000063621, KAT6A, U6, AP3M2, PLAT, IKBKB*	Milk, meat, performance, conformation, weight

a ∆AI, ∆AC, ∆AA, ∆AZ: estimated excess/deficiency of the Iberian, commercial, African and zebu ancestries, respectively. In bold, substantial increase/decrease in ancestry by more/less than two standard deviations (*SD*) from the whole genome mean, respectively.

b Whole genome ancestries obtained with the software LAMP.

**Table 5:** Enriched KEGG signalling pathways for genomic regions under positive selection in Florida Cracker, Senepol and Texas Longhorn breeds

KEGG pathway	Genes	*p*‐value	Fold enrichment
Florida Cracker (CRK)			
bta05031: Amphetamine addiction	*GRIN1, SLC18A2, CAMK2D*	0.007	22.64
bta05030: Cocaine addiction	*GRIN1, SLC18A2*	0.087	20.63
Senepol (SNP)			
bta04060: Cytokine‐cytokine receptor interaction	*IFNAR2, FLT3, LIFR, IFNGR2, IFNAR1*	0.002	8.85
bta04630: Jak‐STAT signalling pathway	*IFNAR2, LIFR, IFNGR2, IFNAR1*	0.004	11.16
bta04620: Toll‐like receptor signalling pathway	*IFNAR2, CD80, IFNAR1*	0.023	12.04
bta04650: Natural killer cell‐mediated cytotoxicity	*IFNAR2, IFNGR2, IFNAR1*	0.028	10.80
bta04380: Osteoclast differentiation	*IFNAR2, IFNGR2, IFNAR1*	0.035	9.43
bta05162: Measles	*IFNAR2, IFNGR2, IFNAR1*	0.038	9.03
bta05164: Influenza A	*IFNAR2, IFNGR2, IFNAR1*	0.056	7.31
bta05168: Herpes simplex infection	*IFNAR2, IFNGR2, IFNAR1*	0.066	6.65
Texas Longhorn (TXL)			
bta04970: Salivary secretion	*CD38, BST1, LYZ*	0.007	21.08
bta04972: Pancreatic secretion	*CD38, BST1, SCTR*	0.010	18.23
bta00760: Nicotinate and nicotinamide metabolism	*CD38, BST1*	0.050	36.46

Estimation of different ancestries using LAMP allocated slightly different contributions to Iberian, commercial, African and zebu genomic components (Table 4), when compared with the Admixture results (Table 1). Several regions under selection in Creole populations showed strong deviations in ancestry contributions (two standard deviations—*SD*—above or below the genomewide average, see Table 4), mostly detecting increases in the zebu component. Florida Cracker and Senepol displayed higher proportions of regions under selection with strong ancestry deviations (54% and 60%, respectively), all involving zebu haplotypes except for one in Florida Cracker with an increase in Iberian ancestry. The two regions showing strong deviations in Texas Longhorn were driven by African ancestry, one of them coupled with a higher zebu component. In Colombian breeds, only one region displayed a clear increase above the genome average, again with zebu ancestry. Regions showing an increase in zebu ancestry have been associated with traits important for tropical adaptation, such as the sleek hair coat (see below), conformation and stature, reproduction (including a region associated with reproduction traits in Tropical Composite bulls) and heat tolerance (Table 4).

**Figure 8:**
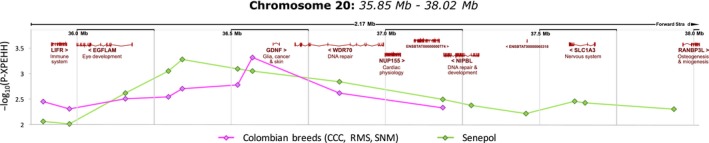
Selection signatures in the BTA20 genomic region shared by the Colombian cluster (Costeño con Cuernos, Romosinuano, San Martinero) and the Senepol breed. Plot of −log_10_(P‐XPEHH) values (*y*‐axis) around loci (*x*‐axis in Mb). Dots mark significant SNPs

The region in BTA20 shared by Colombian (region #11) and Senepol (region #33) populations showed signals of selection with the XP‐EHH analysis and demonstrated a strong increase in zebu ancestry of 38% (more than 6 *SD*) in Colombian breeds and 45% (almost 4 *SD*) in Senepol (Table 4), implying that zebu haplotypes, otherwise representing a small proportion genome wide, are under strong selection in this region and that anthropogenic selection and/or local adaptation rather than genetic drift is driving their presence. Among the genes included in this area, *LIFR* is implicated in immune processes, *NUP155* displays functions in cardiac physiology, *RANBP3L* is implicated in osteogenesis and myogenesis (Chen et al., 2015), and *WDR70* and *NIPBL* are involved in DNA repair processes, highly conserved in nature to remove or tolerate DNA damage caused, among other exogenous factors, by ultraviolet daylight, especially intense in tropical latitudes (Menck & Munford, 2014). This region overlaps with several cattle loci associated with milk traits, mastitis, feed intake, meat attributes, reproduction and weight. Importantly, it also partially overlaps with the region for the slick hair coat, a phenotype that plays an important role in thermotolerance in some tropical Creole breeds, including Senepol and Romosinuano (Flori et al., 2012; Huson et al., 2014). Slick hair coat is characterized by sleek, short hair coupled with increased perspiration. The sleek and shiny properties of this coat may reflect solar radiation more efficiently, and the hair coat thickness and hair weight per unit surface increase heat loss via convection and conduction. As a result, slick animals show lower temperature and respiration rates and an increased production under tropical conditions when compared with normal‐haired individuals (see Flori et al., 2012 for a review). Several studies have associated a region in BTA20 to this phenotype and suggested different candidate genes (*PRLR,* Mariasegaram et al., 2007; *RAI14,* Flori et al., 2012; *SKP2, SPEF2,* Huson et al., 2014). However, the causative mutation is still unknown. Here, the detected region under selection in BTA20 is located slightly downstream (36–38 Mb) compared to the others studies (37–40 Mb), with the most significant SNPs peaking around the *GDNF* gene both in Senepol breed and Colombian group, which included the Romosinuano breed (Figure 8, Table 4, Supporting Information Table S3). A possible explanation for the lack of complete overlap with other studies may be the inclusion in the analyses for the first time of the Iberian populations sharing a common ancestor with Creole cattle in the recent past. The candidate gene for the slick phenotype identified here, the glial cell‐derived neurotrophic factor (*GDNF*), has important roles in skin homeostasis, is involved in the migration and differentiation of melanocytes and shows a strong expression in sebaceous and sweat glands (Adly, Assaf, Pertile, Hussein, & Paus, 2008). It is also implicated in hair follicle morphogenesis and cycling control, increasing the number of the proliferating HF keratinocytes (Adly et al., 2008). However, as in previous studies, the associated SNPs are located in noncoding regions and further studies are needed to narrow down the causative mutation.

Another region in BTA06 showing selection signal with the XP‐EHH methodology in two clusters, Colombian group (region #5) and Texas Longhorn (region #41), has not been associated with any QTL in cattle so far and includes genes such as *C1QTNF7*, related to *Trypanosoma cruzi* cardiomyopathy (Deng et al., 2013), *FBXL5*, which controls iron metabolism processes key for the regulation of reactive oxygen species that augment with the exposure of animals to high environmental temperatures (Paital et al., 2016), *BST1* that has immune functions facilitating pre‐B‐cell growth, and *CD38* that has pleiotropic functions in T‐cell activation (Würsch et al., 2016), social behaviour through its effect on the release of oxytocin (Krol, Monakhov, Lai, Ebstein, & Grossmann, 2015) and cancer. *BST1* and *CD38* are also implicated in salivary and pancreatic secretion and nicotinate and nicotinamide metabolism pathways (Table 5). The genes in this region represent adaptations to new and challenging environments, including immune function, nervous and behavioural processes that may be key for animals to adapt to new environmental conditions, metabolism, high environmental temperatures and diet.

Although the genes included in the region under selection in BTA05 shared by Florida Cracker (region #12) and Texas Longhorn (region #38) and detected with XP‐EHH are mostly uncharacterized novel genes in Ensembl, as well as the antimicrobial agent lysozyme (*LYZ*) and other genes with no clear role in reproduction, this region has been associated with reproduction traits in Tropical Composite bulls. Concordantly, here we found a substantial increase in zebu (by 13%) and African (by 6%) ancestries in the Texas Longhorn, although this was not found in the Florida Cracker. Another region under selection in two clusters, Colombian (region #9) and Texas Longhorn (region #42), was also detected with XP‐EHH methodology and included genes in BTA13 with roles in reproduction (*CFAP61*), neuroendocrine differentiation (*INSM1*), cancer (*RALGAPA2*) or cell cycle (*KIZ*). This region has been previously associated with QTLs related to production traits in cattle (Table 4) and displayed a strong increase in African ancestry (10%, more than 5 *SD*) in Texas Longhorn, but again imperceptible in the Colombian cluster.

Apart from these genomic regions under selection in more than one cluster, we detected signatures of selection associated with a variety of traits (Tables 4, 5, Supporting Information Table S5). These include regions of the genome enriched for genes involved in immune system activation in response to infectious diseases (tick resistance in the Colombian group and Florida Cracker, tuberculosis susceptibility in Florida Cracker and Senepol, mastitis in the Colombian group and Senepol), or enriched immune pathways in Senepol (cytokine–cytokine receptor interaction, Jak‐STAT signalling, Toll‐like receptor signalling, natural killer cell‐mediated cytotoxicity, osteoclast differentiation, and responses to viral diseases ‐measles, influenza A, herpes simplex‐). In addition, we found regions enriched for genes associated with heat tolerance, including regulation of blood pressure and, importantly, thermoregulation in lactating cows exposed to heat stress in the Florida Cracker (region #24). This region in BTA26 showed a strong increase in zebu ancestry (43%, more than three *SD*) and was also implicated in temperament, with the *SLC18A2* gene involved in the dopamine and serotonin pathways associated with temperament in cows (Garza‐Brenner et al., 2017). Phenotypic variation driven by production aims, such as beef or dairy traits, may have had an impact in the genomic areas under selection, highlighted here by the regions detected within QTLs associated with milk and meat production, fatty acid profile, performance, conformation and reproduction.

Finally, we have also validated the signal for the polled locus (Flori et al., 2012; Medugorac et al., 2012) in Senepol (BTA01 region #25), with both XP‐EHH and *F*_ST_ methodologies. This region showed a strong zebu component increase of 47% (almost four *SD* deviations above the genome mean). None of the previously described polled mutations are located in known coding regions. Within our candidate region, the most significant SNPs peaked around three genes, *GART*,* DNAJC28* and *TMEM50B*, none of them with a clear role in polledness ontogenesis. The key immune functions displayed by several genes in this region (*IFNAR2*,* IFNGR2*,* IFNAR1*; Table 4), which could be important in responses against tropical diseases and parasite infections, may distort the signal from the polled locus.

Although *F*_ST_‐ and LD‐based methodologies are widely used, there are other possible factors apart from selection that may mimic the signals obtained, such as demographic events (e.g., the bottlenecks and expansions detected with the ABC and SNeP analyses; Vitti, Grossman, & Sabeti, 2013). Moreover, the use of SNP array markers may underestimate genetic diversity through ascertainment bias, distorting allele frequencies and derived statistics such as LD (Vitti et al., 2013). Also, selection response for complex traits caused by weak selection at many sites across the genome may leave few or no classical signatures (Kemper, Saxton, Bolormaa, Hayes, & Goddard, 2014), reducing the signal obtained. However, other studies on cattle adaptation to new environments (Makina et al., 2015; Porto‐Neto et al., 2014), including tropical adaptation, reported the slick hair coat and QTLs associated with tick resistance, heat tolerance and reproduction in tropical populations.

In conclusion, we compared modern Creole cattle with modern day samples from breeds comprising their putative Iberian ancestors for the first time to reconstruct their demographic history and search for selection signatures enforced by American environments on a small number of founder animals during a brief period of time. We show that despite strong evidence for rapid genomic adaptation to their new tropical environments (e.g., for slick hair coat genes improving thermotolerance), Creole cattle have recently undergone a major decline and will require genetic conservation measures if they are to continue to thrive. The outcomes from this study will contribute to the design of innovative breeding schemes that will include, apart from traditional performance traits, resilience biomarkers, allowing sustainable production in harsh environments and improving sanitary conditions in farms under the ongoing climate changes.

## DATA ARCHIVING STATEMENT

The SNP array data of the 29 animals newly genotyped and the synchronized and filtered 33,342 SNPs data set are available from the Dryad Digital Repository: https://doi.org/10.5061/dryad.g4f4790.

## CONFLICT OF INTEREST

None declared.
