# Supplementary figures and images for: Demography and rapid local adaptation shape Creole cattle genome diversity in the tropics
Source: Evol Appl. 2018 May 18;12(1):105–22. doi: 10.1111/eva.12641 (PMC6304683; doi:10.1111/eva.12641)

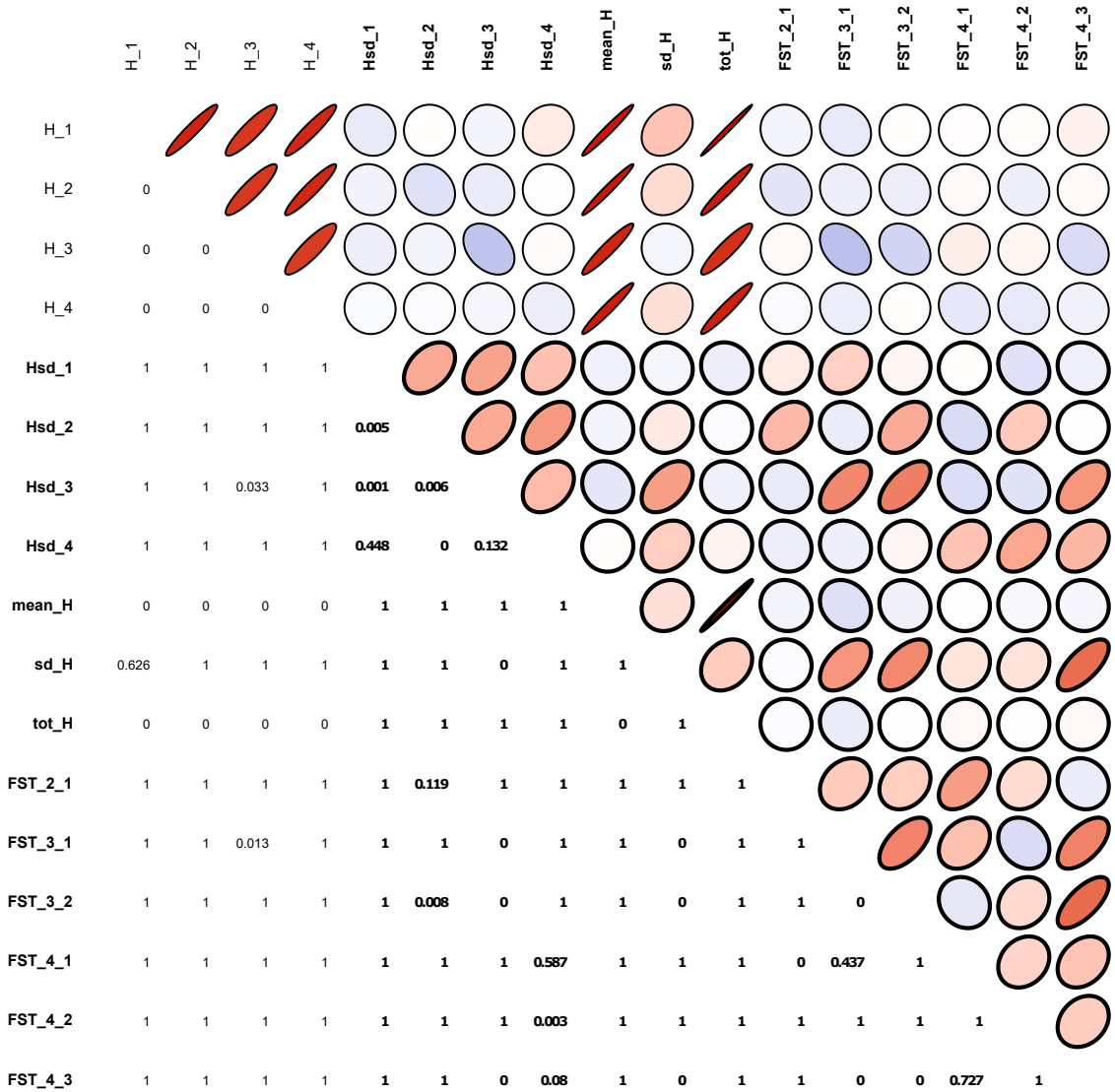

Supplement: Supplementary file 1 [file EVA-12-105-s001.pdf]

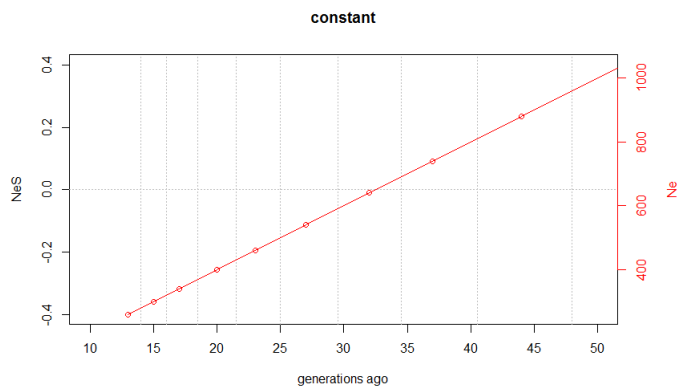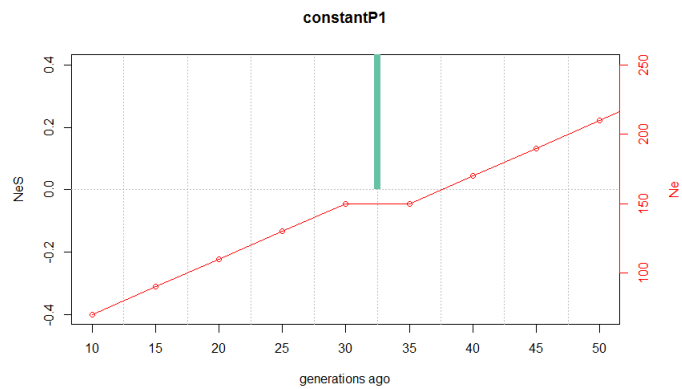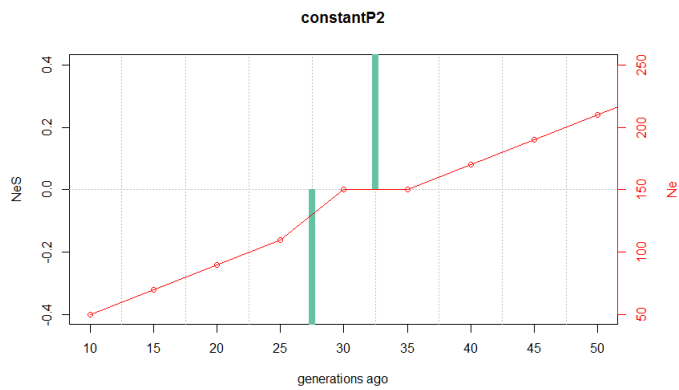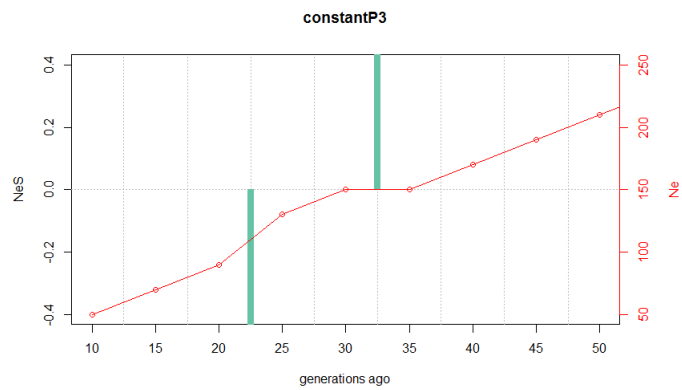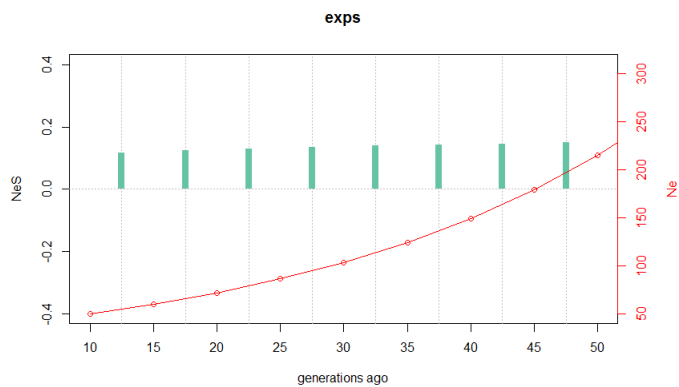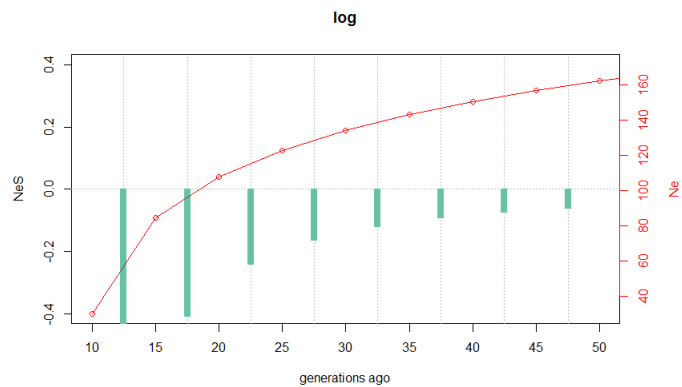

Supplement: Supplementary file 2 [file EVA-12-105-s002.pdf]
