# Supplementary material for: Demography and rapid local adaptation shape Creole cattle genome diversity in the tropics
Source: Evol Appl. 2018 May 18;12(1):105–22. doi: 10.1111/eva.12641 (PMC6304683; doi:10.1111/eva.12641)
Supplement: Supplementary file 3 [file EVA-12-105-s003.pdf]

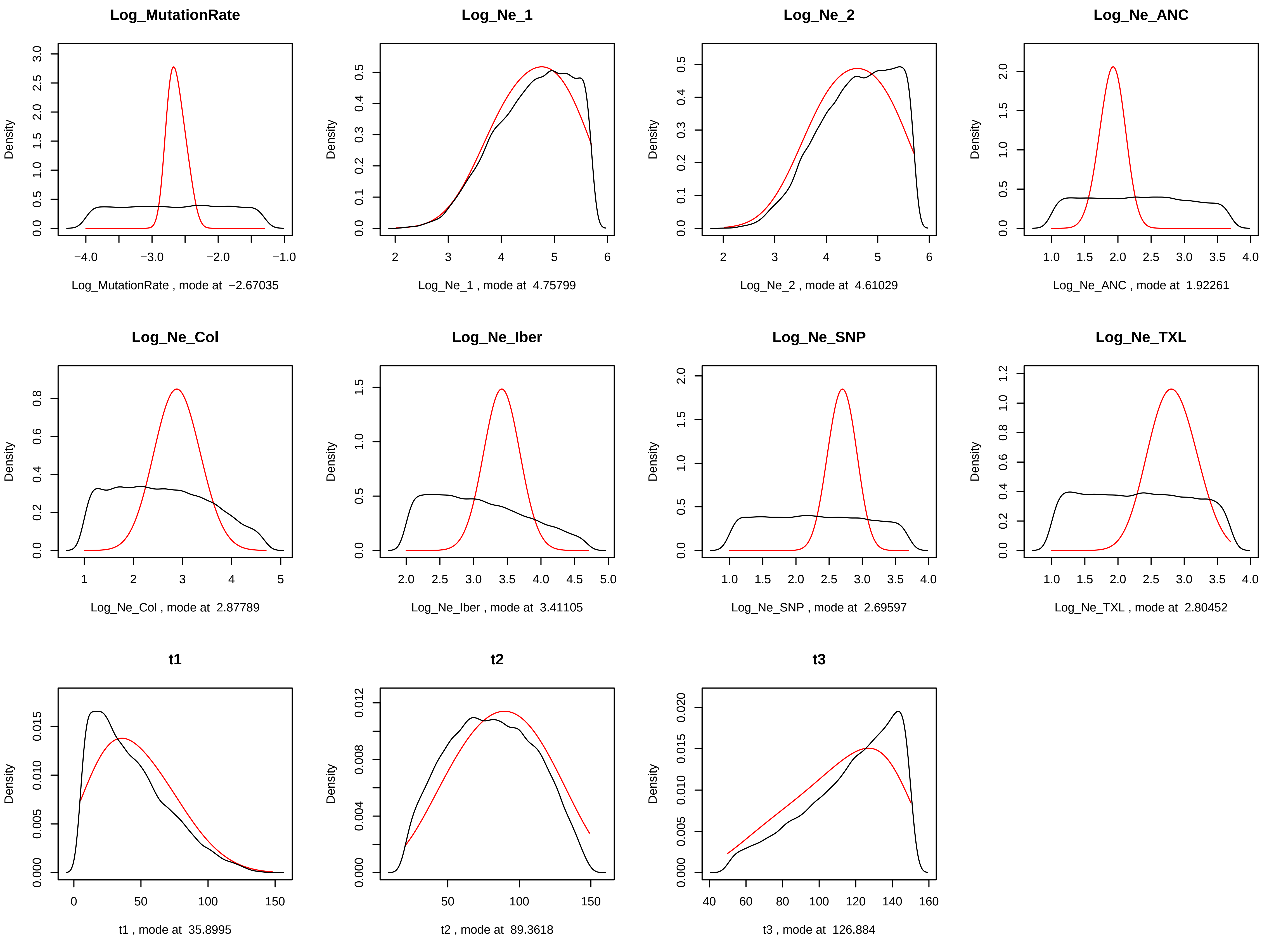
Log_MutationRate
Density
Log_MutationRate , mode at −2.67035
Log_Ne_1
Density
Log_Ne_1 , mode at 4.75799
Log_Ne_2
Density
Log_Ne_2 , mode at 4.61029
Log_Ne_ANC
Density
Log_Ne_ANC , mode at 1.92261
Log_Ne_Col
Density
Log_Ne_Col , mode at 2.87789
Log_Ne_Iber
Density
Log_Ne_Iber , mode at 3.41105
Log_Ne_SNP
Density
Log_Ne_SNP , mode at 2.69597
Log_Ne_TXL
Density
Log_Ne_TXL , mode at 2.80452
t1
Density
t1 , mode at 35.8995
t2
Density
t2 , mode at 89.3618
t3
Density
t3 , mode at 126.884
