# Supplementary material for: Demography and rapid local adaptation shape Creole cattle genome diversity in the tropics
Source: Evol Appl. 2018 May 18;12(1):105–22. doi: 10.1111/eva.12641 (PMC6304683; doi:10.1111/eva.12641)
Supplement: Supplementary file 4 [file EVA-12-105-s004.pdf]

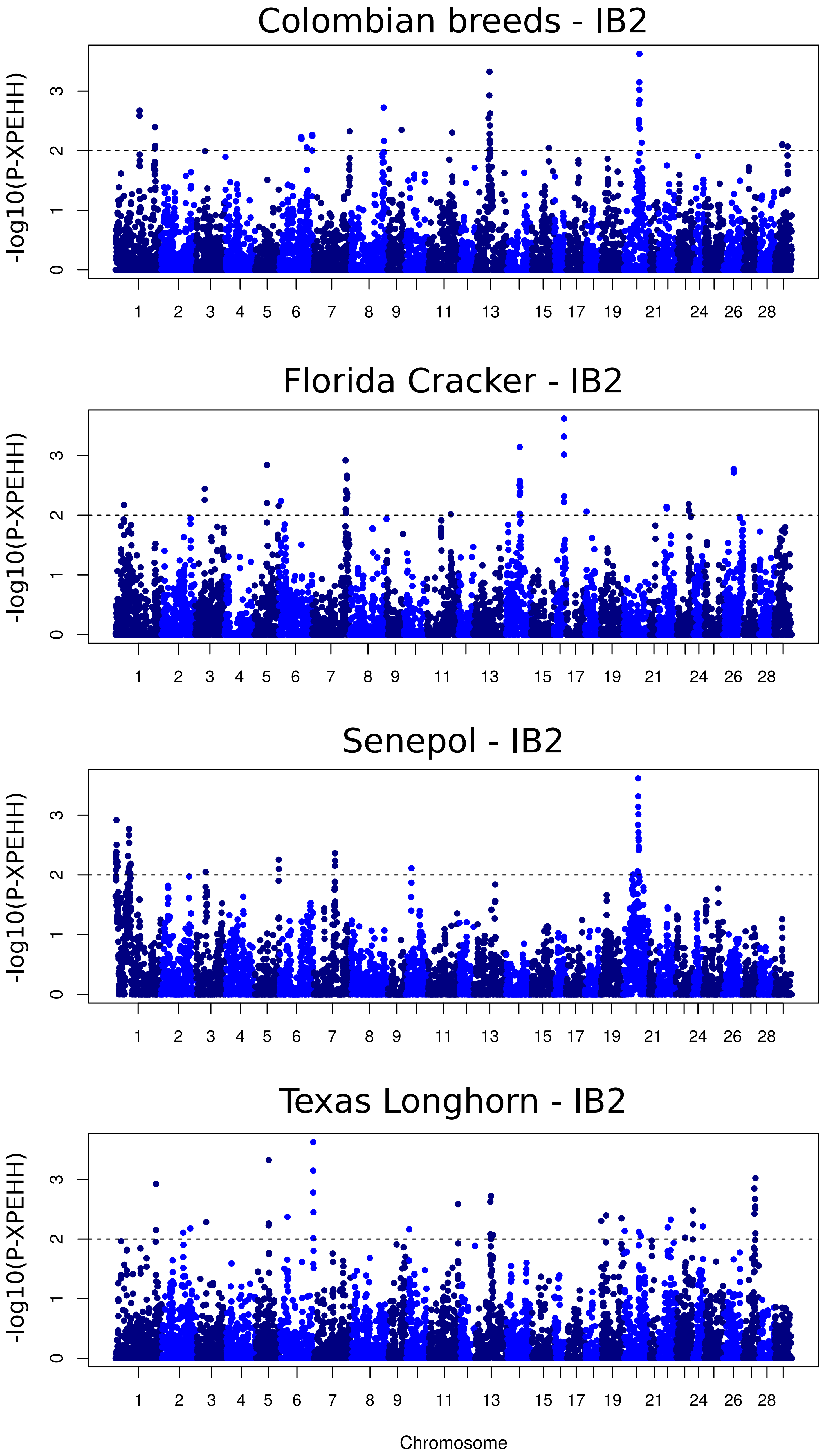
Colombian breeds - IB2
Florida Cracker - IB2
Senepol - IB2
Texas Longhorn - IB2
-log10(P-XPEHH)
0
1
2
3
1 2 3 4 5 6 7 8 9 11 13 15 17 19 21 24 26 28
Chromosome

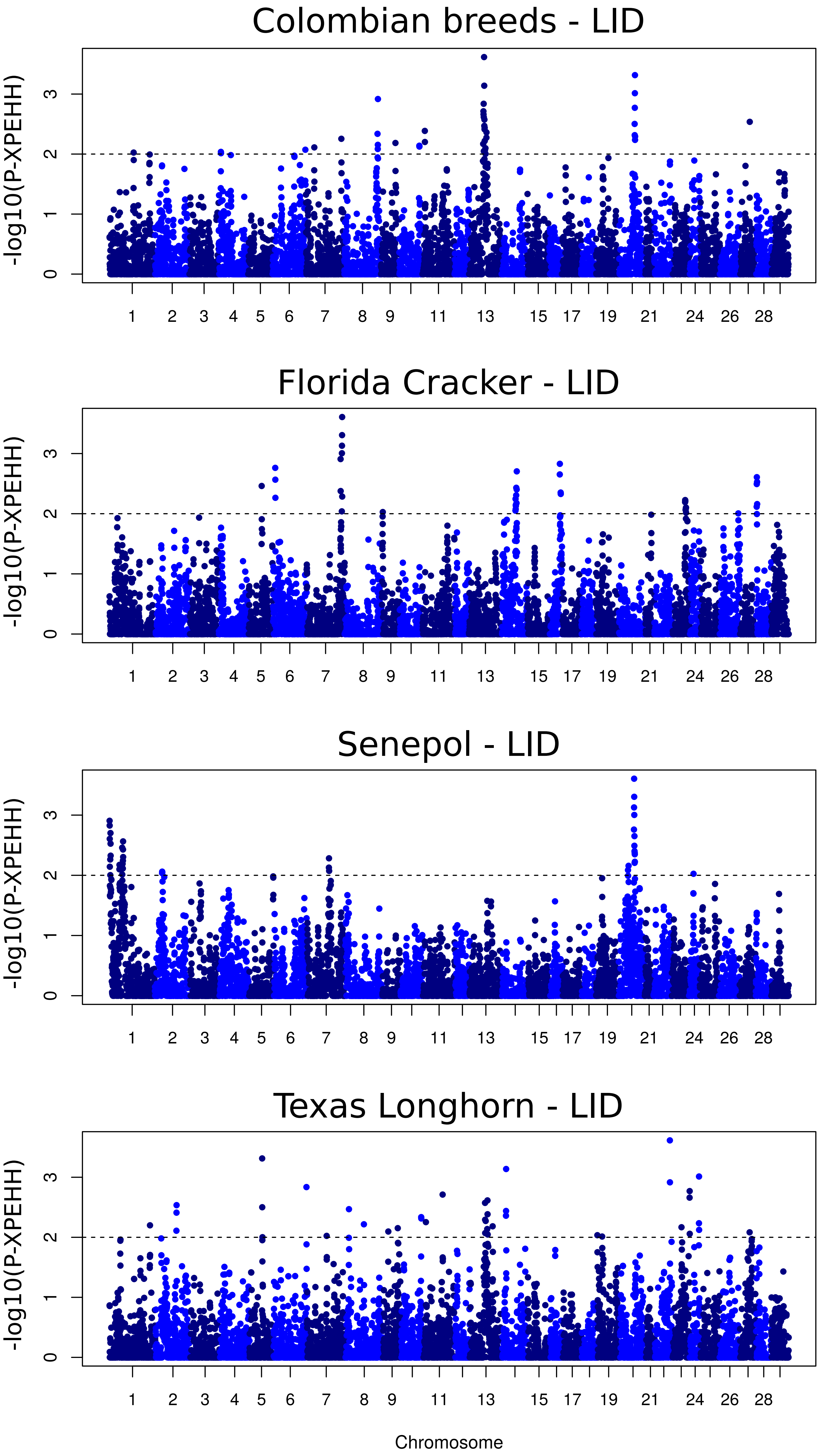
Colombian breeds - LID
-log10(P-XPEHH)
0
1
2
3
1
2
3
4
5
6
7
8
9
11
13
15
17
19
21
24
26
28
Florida Cracker - LID
-log10(P-XPEHH)
0
1
2
3
1
2
3
4
5
6
7
8
9
11
13
15
17
19
21
24
26
28
Senepol - LID
-log10(P-XPEHH)
0
1
2
3
1
2
3
4
5
6
7
8
9
11
13
15
17
19
21
24
26
28
Texas Longhorn - LID
-log10(P-XPEHH)
0
1
2
3
1
2
3
4
5
6
7
8
9
11
13
15
17
19
21
24
26
28
Chromosome
