# Supplementary material for: Demography and rapid local adaptation shape Creole cattle genome diversity in the tropics
Source: Evol Appl. 2018 May 18;12(1):105–22. doi: 10.1111/eva.12641 (PMC6304683; doi:10.1111/eva.12641)
Supplement: Supplementary file 5 [file EVA-12-105-s005.pdf]

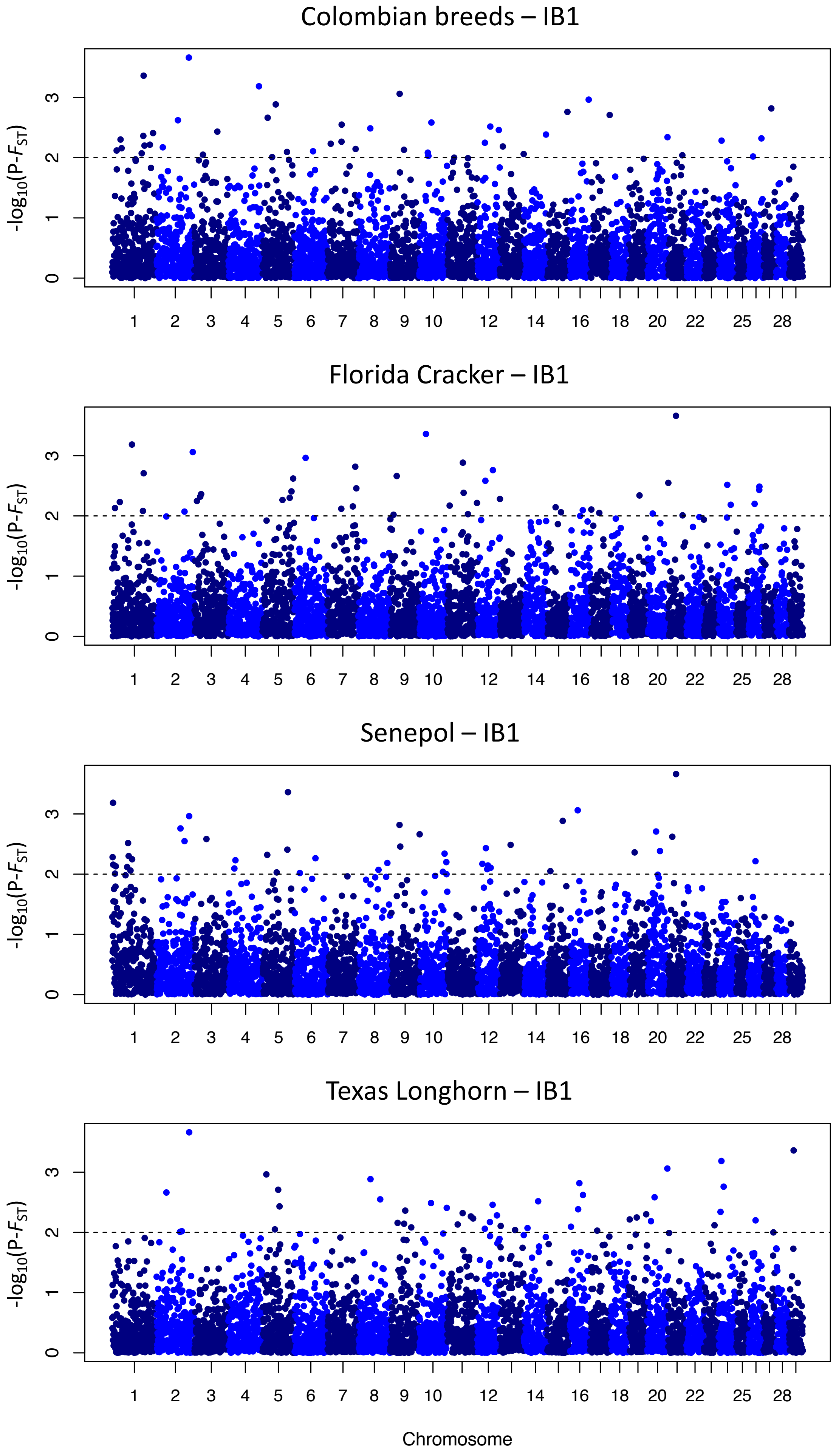
Colombian breeds – IB1
Florida Cracker – IB1
Senepol – IB1
Texas Longhorn – IB1
-log10(P-FST)
0
1
2
3
1
2
3
4
5
6
7
8
9
10
12
14
16
18
20
22
25
28
Chromosome

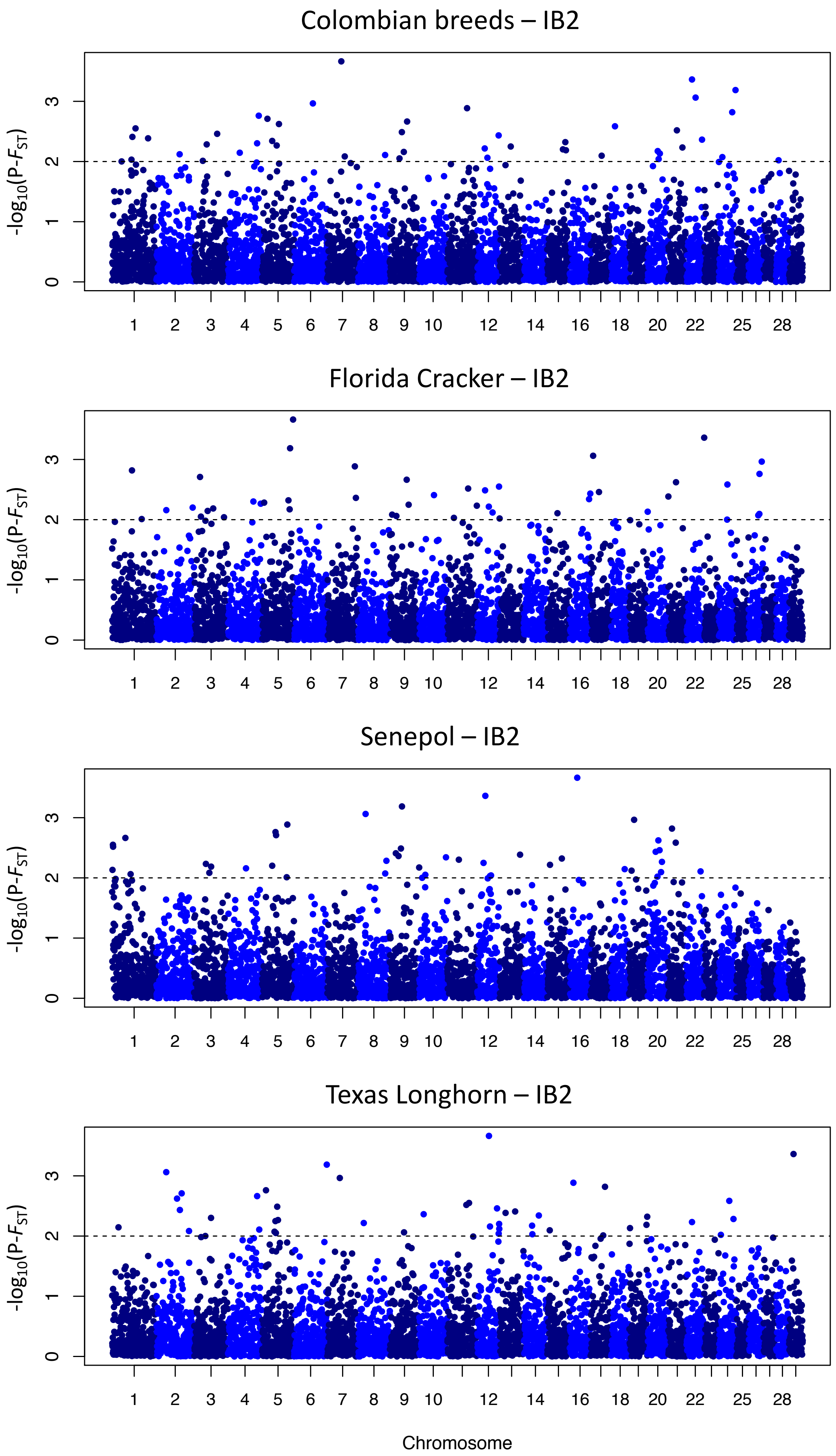
Colombian breeds – IB2
Florida Cracker – IB2
Senepol – IB2
Texas Longhorn – IB2
-log10(P-FST)
0
1
2
3
1
2
3
4
5
6
7
8
9
10
12
14
16
18
20
22
25
28
Chromosome

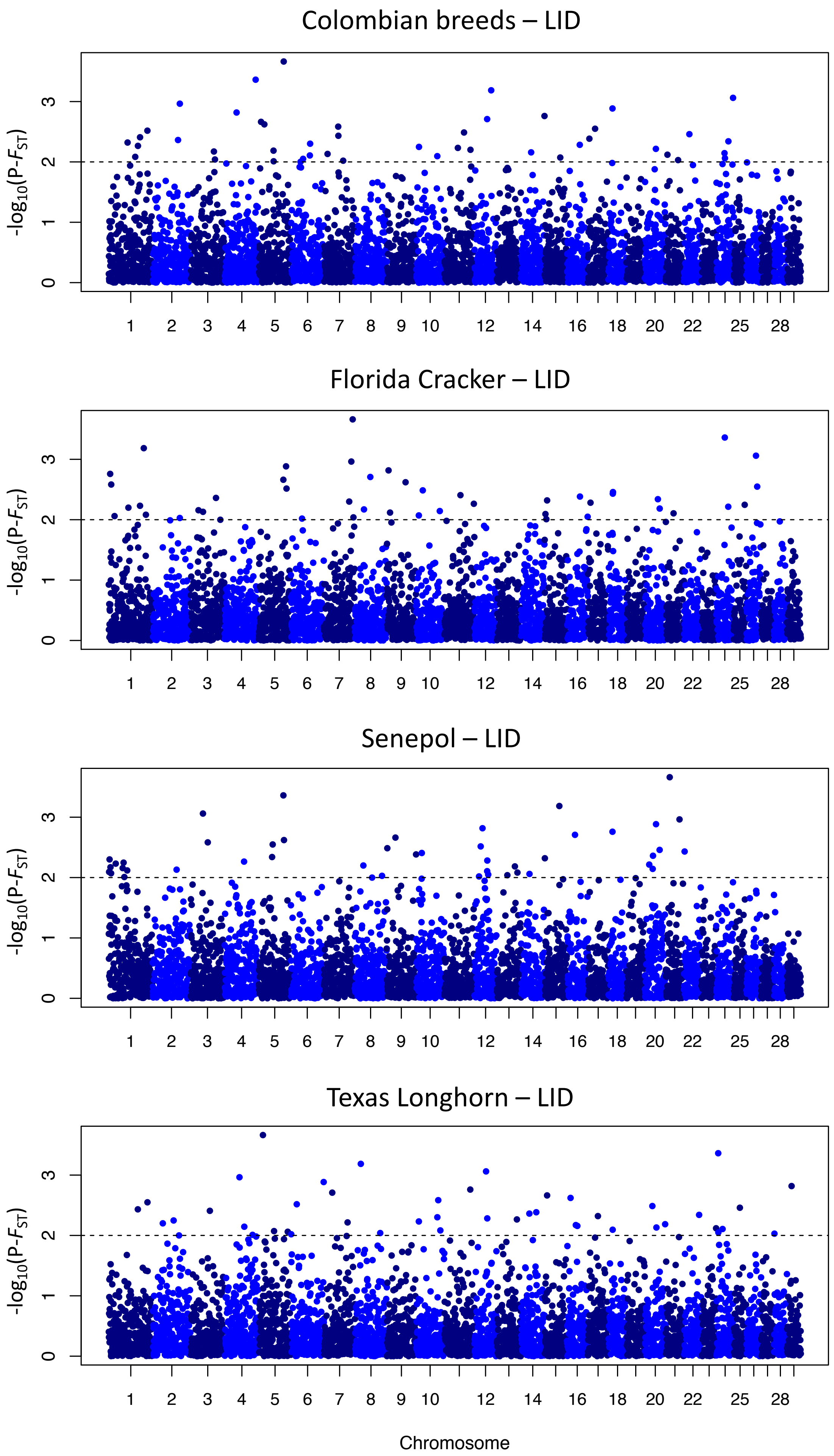
Colombian breeds – LID
Florida Cracker – LID
Senepol – LID
Texas Longhorn – LID
$-\log_{10}(P\text{-}F_{ST})$
0
1
2
3
1
2
3
4
5
6
7
8
9
10
12
14
16
18
20
22
25
28
Chromosome
